# Ultra-Processed Foods and the Cardiovascular-Kidney-Metabolic Continuum: Integrating Epidemiological, Multi-Omics, and Translational Evidence

**DOI:** 10.3390/nu18071039

**Published:** 2026-03-25

**Authors:** Saiful Singar, Amirhossein Ataei Kachouei, Leandro Lantigua-Somoano, David Manley, Anthony Cardinale, Muhammad Zulfiqah Sadikan, Saurabh Kadyan, Donya Shahamati, Lorena Dias, Amber Wood, Cinthia Chavarria, Sara K. Rosenkranz, Neda S. Akhavan

**Affiliations:** 1 EVMS Division of Endocrine and Metabolic Disorders, Macon & Joan Brock Virgina Health Sciences at Old Dominion University, Norfolk, VA 23510, USA; ssingar@odu.edu; 2 Faculty of Life Sciences: Food, Nutrition, and Health, University of Bayreuth, 95326 Kulmbach, Germany; amirhossein.ataei-kachouei@uni-bayreuth.de; 3 Department of Health, Nutrition, and Food Sciences, Florida State University, Tallahassee, FL 32306, USA; lsl22b@fsu.edu (L.L.-S.); dsm22@fsu.edu (D.M.); 4 Department of Chemistry and Biochemistry, Florida State University, Tallahassee, FL 32306, USA; amc21t@fsu.edu; 5 Faculty of Pharmacy and Health Sciences, Universiti Kuala Lumpur Royal College of Medicine Perak, Ipoh 30450, Perak, Malaysia; mzsadikan@yahoo.com; 6 Dairy Microbiology Division, ICAR-National Dairy Research Institute, Karnal 132001, Haryana, India; kadyan.saurabh3@gmail.com; 7 Department of Kinesiology and Nutrition Sciences, University of Nevada, Las Vegas, NV 89154, USA; shahamat@unlv.nevada.edu (D.S.); diasl2@unlv.nevada.edu (L.D.); wooda13@unlv.nevada.edu (A.W.); chavac4@unlv.nevada.edu (C.C.); sara.rosenkranz@unlv.edu (S.K.R.)

**Keywords:** cardiovascular-kidney-metabolic syndrome, ultra-processed foods, NOVA classification, multi-omics, metabolomics, lipidomics, proteomics, gut microbiome, inflammation

## Abstract

Cardiovascular-kidney-metabolic (CKM) syndrome integrates excess adiposity, metabolic dysfunction, kidney impairment, subclinical cardiovascular diseases, and clinical events along a staged continuum that invites unified prevention and treatment. Ultra-processed foods (UPFs) are a complex, high-prevalence exposure that may influence risk across CKM stages through nutrient profiles, additives, processing-induced compounds, and packaging-related contaminants. This review synthesizes epidemiologic, mechanistic, and translational evidence with attention to exposure definition and analytic rigor. We summarize NOVA-based UPF operationalization across dietary assessment tools, highlighting misclassification of mixed dishes, brand heterogeneity, and energy under-reporting, and we propose further examination of energy-adjusted models, calibration, and harmonized metrics. Observational studies consistently associate higher UPF intake with adiposity, diabetes, chronic kidney disease, cardiovascular events, and mortality, with modest to moderate effect sizes that are heterogeneous across populations. Mechanistic data from metabolomics, lipidomics, proteomics, and the gut microbiome converge on pathways of inflammation, lipid metabolism, oxidative and metabolic stress, and intestinal barrier dysfunction; in selected cohorts, multi-omics modules account for a substantial minority of UPF-outcome associations. We outline quality-control pipelines, batch-effect prevention/correction, and multiple-testing control necessary for reproducible diet-omics. Translationally, targeted lipidomic and proteomic panels show promise for CKM risk stratification and monitoring but require validation, clinical thresholds, and guideline endorsement. Equity and global context, including differences in product mix, food systems, and care capacity, modify population impact. We conclude with a research agenda prioritizing harmonized exposure metrics, error-aware modeling, standardized multi-omics workflows, and adequately powered, stage-specific interventions capable of testing mediation and prognostic utility.

## 1. Introduction

**Figure 1:**
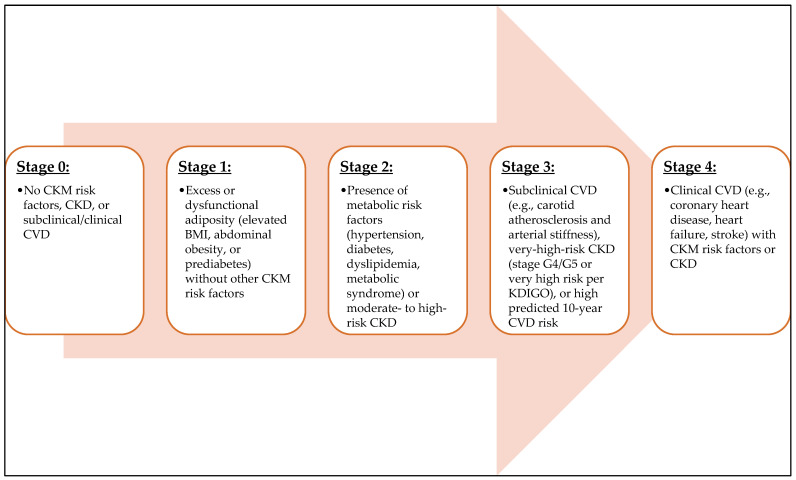
Cardiovascular-Kidney-Metabolic (CKM) syndrome staging (AHA 2023). CKD: chronic kidney disease, CVD: cardiovascular disease, BMI: body mass index, KDIGO: Kidney Disease: Improving Global Outcomes.

Cardiovascular-kidney-metabolic (CKM) syndrome is increasingly recognized as a unified clinical construct that captures the tight bidirectional links among excess or dysfunctional adiposity, metabolic dysregulation, chronic kidney disease (CKD), and cardiovascular diseases (CVD). The American Heart Association (AHA) formalized this syndrome with a staging framework (Figure 1) that aligns risk assessment and care across the life course and specialties. The framework emphasizes common pathophysiology and shared determinants, encouraging integrated prevention and treatment strategies that transcend traditional silos of obesity, diabetes, nephrology, and cardiology [1,2,3].

Against this backdrop, ultra-processed foods (UPFs) warrant attention as a complex dietary exposure with relevance across all stages of CKM. Major classification systems, most prominently NOVA, characterize UPFs as industrial formulations of ingredients that are largely extracted, refined, or synthesized, typically combined with cosmetic additives to enhance palatability and shelf stability [4]. As a class, UPFs tend to be energy-dense and hyperpalatable, with higher amounts of refined starches, added sugars, sodium, and certain fats, and lower amounts of intact fiber and micronutrients. These features align with weight gain, insulin resistance, hypertension, and dyslipidemia, all of which are central drivers of CKM progression [5,6,7,8]. Processing-related features extend beyond nutrient composition to include food additives, such as emulsifiers and certain artificial sweeteners, which can perturb gut barrier function and microbiota, with downstream consequences for metabolic and inflammatory signaling implicated in CKM risk [9,10]. UPFs can also serve as sources of food-contact chemicals and processing by-products, including phthalates and per- and polyfluoroalkyl substances, that are associated with adverse cardiometabolic profiles and kidney-relevant toxicities, adding another potential mechanism that may be important in CKM pathogenesis [11,12,13].

Positioning CKM as a single continuum clarifies why UPFs may influence risk at multiple points along the continuum. Diets high in UPFs can promote excess adiposity early in the trajectory, amplify intermediate metabolic perturbations such as glucose intolerance, dyslipidemia, and low-grade inflammation, and exacerbate kidney injury and subclinical CVD, thereby increasing the likelihood of clinical events later in the progression of CKM. Conversely, reducing UPF exposure appears to shift systems-level biology toward lower risk. Emerging human evidence suggests that altering UPF intake can modulate circulating metabolite profiles and the gut microbiome composition, providing measurable biological signatures that align with CKM pathways and may complement traditional risk factors in staging and monitoring [6,9,10].

While the epidemiological evidence linking UPFs to adverse health outcomes is vast, with recent umbrella reviews synthesizing over 40 pooled analyses encompassing nearly 10 million individuals, the application of high-throughput omics technologies to this field remains a nascent but rapidly accelerating frontier. Currently, the literature features a select but growing number of dedicated omics investigations, primarily dominated by single-layer untargeted metabolomics in large observational cohorts. Recent high-quality publications have significantly advanced the UPF-CKM paradigm, yet they primarily fall into three distinct categories: (1) macroscopic epidemiological umbrella reviews that quantify population-level hazard but lack mechanistic depth; (2) clinical narrative reviews that describe broad pathophysiological concepts (e.g., meta-inflammation) without actionable molecular readouts; and (3) primary observational cohorts focused exclusively on single-layer untargeted metabolomics.

The novelty of this manuscript lies in its synthesis of these previously siloed domains into a singular, actionable framework. Unlike recent umbrella and narrative reviews, this work provides high-resolution, quantifiable multi-omics signatures (spanning lipidomics, proteomics, and the gut microbiome) rather than abstract mechanisms, mapping these molecular readouts directly onto the AHA’s CKM staging. Furthermore, unlike recent single-layer metabolomic studies, this review moves beyond observational hazard identification by providing a novel methodological blueprint for future randomized controlled trials. By detailing specific sample size matrices, temporal sampling guidelines, and rigorous bioinformatic quality-control pipelines (e.g., batch-effect correction and false discovery rate controls) required for diet-omics, this review uniquely establishes the technical architecture necessary to transition the field from observational correlations to molecularly precise clinical interventions.

## 2. Literature Search Strategy

To synthesize the current epidemiological, multi-omics, and translational evidence regarding UPFs and CKM syndrome, a comprehensive literature search was conducted. We searched for major electronic databases, including PubMed, Embase, Cochrane Library, and Web of Science, for peer-reviewed articles published up to August 2025. The search strategy utilized combinations of the following primary keywords and Medical Subject Headings (MeSH): “ultra-processed foods”, “NOVA classification”, “cardiovascular-kidney-metabolic syndrome”, “multi-omics”, “metabolomics”, and “gut microbiome”. The Boolean search string utilized during our database queries included *(“ultra-processed food” OR “UPF” OR “NOVA classification”) AND (“cardiovascular-kidney-metabolic syndrome” OR “CKM syndrome” OR “metabolic syndrome” OR “chronic kidney disease” OR “cardiovascular disease”) AND (“multi-omics” OR “metabolomics” OR “proteomics” OR “lipidomics” OR “gut microbiome”)*.

Article selection was restricted to studies published in English that involved human cohorts or clinical trials and directly investigated the physiological or epidemiological impact of UPF exposure on cardiometabolic or renal outcomes. In addition to database searches, the reference lists of recent umbrella reviews, systematic reviews, and meta-analyses were manually screened to identify any further relevant high-quality publications.

## 3. Defining and Measuring the UPF Exposure

**Figure 2:**
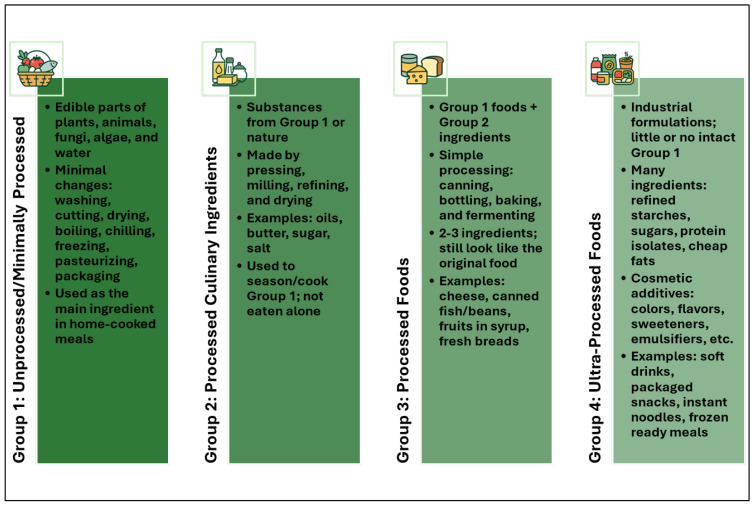
NOVA classification.

In epidemiology, the intake of UPFs is most commonly operationalized using the NOVA framework, which categorizes foods into four groups based on the extent and purpose of industrial processing, as depicted in Figure 2. Group 4 denotes ultra-processed products formulated from extracted or synthesized ingredients and cosmetic additives [14,15]. In practice, NOVA coding is applied to data from 24 h dietary recalls, food records, and food-frequency questionnaires and sometimes supplemented by barcode or ingredient-list databases when brand-specific information is available. Across tools, inter-rater reliability for the NOVA assignment is generally moderate to good, and construct validity is acceptable; however, performance depends on the granularity of the underlying dietary instrument and the completeness of product information [6,16,17,18,19,20,21,22,23,24]. Item-level 24 h recalls enable finer differentiation of processing levels but are vulnerable to uncertainty for mixed dishes and items lacking full ingredient descriptors (concordance ≈ 88%, Cohen’s κ ≈ 0.75). NOVA adaptations for food-frequency questionnaires help rank exposure in large cohorts but lose detail to separate composite foods (ICC 0.85–0.94), which can weaken differences between processing categories. Barcode- or ingredient-based classification improves labeling for clear packaged items but remains inconsistent for ambiguous products and reformulated brands (Fleiss’ κ ≈ 0.32–0.34), especially with incomplete or region-specific ingredient lists [15,25,26,27,28,29].

Misclassification sources include coding mixed dishes at the dish level rather than by components, confusing home-cooked with ready-to-eat options, and “health-positioned” products like whole-grain breads with minor additives that place them in NOVA group 4 despite good nutrient profiles. Conversely, minimally processed foods with poor nutrient profiles may avoid UPF classification when judged only by processing level. These ambiguities cause misclassification errors that bias results toward no effect and can lead to differential errors if linked to health status, brand use, or cultural food preferences [15,25,26,27].

Energy under-reporting further complicates UPF exposure estimates. Under-reporting is common in self-reported dietary data, increases with higher BMI, and may differ for ultra-processed items due to social desirability and recall cues. The net effect is systematic underestimates of both total energy and the UPFs, with consequent attenuation of exposure-outcome associations. Methodological remedies include excluding implausible reporters using established cut-offs, adjusting for total energy intake, and conducting sensitivity analyses comparing results across alternative energy-adjustment strategies. These approaches improve internal validity but do not eliminate bias when misreporting varies by health status or when underreporting influences weight change [21,23,24,25,26].

For cross-study synthesis, the percentage of energy from UPFs is the most comparable metric because it adjusts intake relative to total energy, minimizes confounding from energy needs and reporting errors, and aligns with common practices in large cohorts and pooled analyses. Density models and the residual method estimate ultra-processed intake separately from total calories, which is essential for comparing populations with different energy needs or under-reporting tendencies. Other metrics like servings per day or grams of NOVA group 4 foods can be useful in specific situations but are more affected by energy density, recipe variety, and portion-size errors, which hinder comparison across tools and settings. Even with energy-adjusted models, residual confounding and measurement inaccuracies remain, especially due to ongoing classification ambiguities and reporting biases. This review interprets UPF-CKM relationships by emphasizing the percentage of energy from UPF based on validated NOVA coding, acknowledging some residual bias despite best-practice efforts and adjustments [15,25,26,27,29,30].

## 4. Epidemiologic Signal Linking UPFs to CKM-Relevant Outcomes

Large prospective cohorts and quantitative syntheses consistently show that higher intake of UPFs is linked to an increased risk of poorer CKM outcomes across various populations. Recent umbrella reviews and meta-analyses that combine dozens of cohort studies generally report similar findings for incident CVDs, type 2 diabetes, CKD, metabolic syndrome, and all-cause mortality. These estimates are consistent across European, North American, and Latin American populations, as well as across different ages and sexes (Table 1) [31,32,33,34]. These syntheses typically identify graded dose–response relationships when exposure is modeled as a percentage of total energy from UPFs, supporting a positive association between the level of ultra-processing in the diet and CKM risk [31,33].

Across outcomes, effect sizes are modest to moderate but consistent. For type 2 diabetes, multiple cohorts and meta-analyses have documented dose–response associations, including pooled estimates of a 12 percent higher relative risk per 10 percent increase in energy from UPFs, with confirmatory cohort-specific findings, such as a hazard ratio of 1.17 for the highest versus the lowest exposure in The European Prospective Investigation into Cancer and Nutrition (EPIC) prospective study after multivariable adjustments [31,38]. For CVD, large cohorts and pooled analyses similarly indicate a higher incident risk with higher UPF intake, with summary relative risks in the low-to-mid teens per 10% energy increment, consistent with a small but meaningful shift in population risk [25,31,33,35]. For CKD, prospective data from the Lifelines Cohort Study and complementary meta-analytic summaries suggest elevations in risk of 13 to 27 percent comparing higher with lower intake, with some evidence that the gradient is steeper in earlier stages of kidney dysfunction [31]. For metabolic syndrome, cohort data from The Brazilian Longitudinal Study of Adult Health (ELSA-Brasil) indicate approximately a 1.3-fold higher incidence at higher consumption levels, aligning with meta-analytic signals implicating UPF intake in the clustering of metabolic risk factors salient to CKM staging [33,39]. Mortality signals are directionally similar, with higher UPF intake predicting higher all-cause mortality in pooled and cohort-specific analyses, reinforcing clinical relevance beyond intermediate risk factors [31,34,35].

Heterogeneity is persistent throughout the evidence based on UPF intake and associated diseases and conditions, but it is explainable. Variation arises from exposure measurement, geographic dietary patterns, and adjustment sets (e.g., overall nutritional/dietary quality indices). Several cohorts have demonstrated that associations attenuate but generally persist after controlling for global diet quality indices and specific nutrients, indicating partial independence of ultra-processing from conventional nutritional confounding [35,41,42,43,44]. Variations in how mixed dishes and ambiguous products are coded, along with differences in energy under-reporting, contribute to variability between studies. This is evident in the wider confidence intervals for certain outcomes and in the outcome-specific certainty ratings across umbrella reviews [31,32]. Importantly, substitution and isocaloric modeling strategies show that replacing ultra-processed foods with minimally processed options is linked to a lower risk of incident CKM conditions and mortality, supporting a causal interpretation by placing the exposure in realistic dietary contexts [25,31,35].

## 5. Multi-Omics as Readouts and Mediators of UPF Effects in CKM

Multi-omics provides both quantitative readouts of systems biology disturbed by UPF intake and plausible mediators linking this exposure to CKM outcomes. Across different groups and analytical methods, metabolomic and proteomic modules indicating low-grade inflammation, lipid metabolism, and metabolic stress are consistently part of the pathway connecting UPF intake to negative clinical outcomes. Formal mediation analyses indicate that these omics layers account for a substantial minority of the total association, with estimates typically ranging from approximately 20% to 43%, depending on the outcome, biomarker panel, and modeling strategy. For instance, in large biobank settings, inflammatory protein signatures play a significant role in mediating the relationship between UPF and cardiovascular mortality, accounting for up to 22%. Likewise, composite metabolomic scores from plasma and urine also mediate parts of the links with incident metabolic and vascular outcomes [45]. These findings support the interpretation that UPF-related biological perturbations are not merely correlates of intake but encode intermediate mechanisms along the CKM continuum [45,46,47,48,49].

Two features make inflammation- and lipid-centric omics modules the most reproducible signals to date [27,49,50]. Initially, they converge across various platforms and populations: targeted and untargeted metabolomics consistently identify branched-chain amino acids, acylcarnitines, and other lipid-related intermediates indicating disrupted mitochondrial oxidative metabolism. Simultaneously, proteomic panels detect cytokines, acute-phase reactants, complement, and coagulation factors that reflect inflammation in adipose tissue and blood vessels [30,50,51]. Secondly, these modules demonstrate external validity by being connected not only to UPF exposure but also to downstream phenotypes like insulin resistance, dyslipidemia, subclinical atherosclerosis, kidney function decline, and clinical cardiovascular events. The associated risk gradients remain significant even after multivariable adjustment. Together, these properties reduce noise from instrument-specific artifacts and support transportability across cohorts, which is essential for synthesis and for staging CKM risk with biologically anchored measures [45,46,47].

It is equally important to recognize the limits of current mediation. The remaining unexplained part of the UPF-outcome link probably indicates other biological factors that current panels only partly detect. These include metabolites from gut microbiome, hormonal and autonomic stress pathways, and kidney and blood vessel processes; direct dietary impacts such as energy density, sodium content, and food structure; and lingering confounding due to misclassification of exposures and reporting inaccuracies. These considerations highlight the need for integrated models that combine high-resolution omics data with thorough exposure assessment and careful covariate control, while recognizing that even well-designed mediation analyses will only uncover part of a complex causal network. Framed within the AHA’s CKM staging, multi-omics can therefore serve as both mechanistic intermediates and quantitative readouts that map where and how UPFs perturb the continuum from excess adiposity and metabolic risk to kidney dysfunction, subclinical cardiovascular disease, and hard clinical outcomes [5,45,46].

Multi-omics are treated as both mechanistic readouts and statistical mediators by integrating heterogeneous layers into lower-dimensional constructs that can be related to UPF exposure and CKM outcomes. Latent-factor methods, like multi-omics factor analysis, generate cross-omics components that identify shared biological variations across metabolomics, lipidomics, proteomics, and epigenomics. This enhances the signal-to-noise ratio compared to analyzing single layers and helps uncover molecular subtypes that correspond with CKM severity and trajectories [52,53,54,55]. Complementary network strategies, such as weighted co-expression or co-abundance networks and sparsity-inducing integrative models like sparse partial least squares, group correlated features into modules that reflect pathways involved in inflammation, lipid metabolism, and metabolic stress. These modules provide stable units of analysis that are portable across cohorts and better reflect pathway-level heterogeneity than individual biomarkers, thereby reducing the multiple-testing burden and enhancing biological interpretability [52,53,55,56].

The incremental value of these integrative models over traditional risk factors is twofold. First, latent factors and network modules enhance discrimination and risk stratification when combined with age, adiposity, blood pressure, glycemia, lipids, and kidney function, yielding consistent gains in large cohorts and biobanks through cross-validated machine learning and reclassification metrics [55,57,58]. Second, these models offer mechanistic specificity that augments absolute risk estimation frameworks by anchoring prediction in biologically coherent axes of inflammation and metabolism, a priority articulated by recent scientific statements on integrated cardiovascular risk assessment [1]. In nutritional epidemiology, poly-metabolite scores that summarize diet-linked features demonstrate how omics composites can enhance exposure measurement and strengthen associations with CKM endpoints compared to single metabolites, while retaining transportability across platforms and study designs [30,55].

## 6. The Gut Microbiome as an Interface Between UPFs and CKM Physiology

The gut microbiome serves as a dynamic interface through which UPFs influence host physiology across the CKM stages. Narrative syntheses and empirical studies converge on three interrelated domains. First, microbial signaling integrates dietary cues into host metabolic and inflammatory pathways. Cross-sectional and interventional work link higher UPF consumption to shifts in taxa and metabolites consistent with insulin resistance and low-grade inflammation, including reductions in short-chain fatty acid production and alterations in tryptophan catabolism, detectable within days of dietary change [9,59]. These compositional and functional changes co-segregate with cardiometabolic risk traits and persist after adjustment for conventional dietary quality indices, underscoring biology beyond nutrient totals alone [60,61]. Second, diet-microbe co-metabolism provides plausible mediating chemistry that maps onto CKM pathophysiology. Reviews and cohort analyses suggest that microbially derived metabolites, such as short-chain fatty acids and indole derivatives, play a role in regulating vascular, renal, and metabolic functions, providing a mechanistic pathway through which processing-related features of UPFs can influence blood pressure, glycemic control, dyslipidemia, and kidney function, thereby anchoring the AHA staging framework [62,63,64]. Third, the barrier function emerges as a proximal target of ingredients commonly found in UPFs. Controlled feeding studies and ex vivo work demonstrate that several emulsifiers compromise epithelial integrity, alter mucus structure, and reconfigure microbial communities, with concomitant inflammatory signaling that provides a biologically coherent link to CKM outcomes [65,66,67,68].

Narrative reviews focused on ultra-processed diets describe consistent associations with dysbiosis signatures and barrier dysfunction, integrating human, animal, and in vitro findings to argue that additives and processing artifacts act in concert with nutrient profiles to reshape the gut ecosystem [10,63]. Population studies comparing individuals across gradients of UPF intake report reproducible differences in microbiome composition and diversity that correlate with adiposity and metabolic traits, reinforcing epidemiologic signals for diabetes, chronic kidney disease, and cardiovascular events [9,61]. Short-term diet-switch experiments suggest that microbial community structure and tryptophan-derived metabolites respond rapidly to dietary patterns characterized by fast food versus Mediterranean-style choices, aligning exposure changes with modifiable biochemical readouts relevant to CKM staging [59]. Complementing these observations, randomized trials directly testing emulsifier exposure demonstrate increased intestinal permeability and shifts in microbiota and metabolome profiles compared to baseline, a low-emulsifier diet, or a placebo control, thereby strengthening causal inference for barrier disruption as one mechanistic lever by which UPFs can aggravate CKM risk [65,66,68].

Within the CKM framework, the microbiome can function as both a mediator and an effect modifier. Mediation is supported by evidence that microbiome-linked metabolites track UPF intake and predict downstream phenotypes that define CKM staging, including blood pressure, glycemia, lipid profiles, albuminuria, and subclinical cardiovascular disease [62,64]. Modification is plausible because baseline adiposity, kidney function, and medication use can alter the microbiome’s responsiveness to dietary perturbation and the host’s sensitivity to microbial products, creating heterogeneity in risk transmission even under similar exposure levels [63,64]. Taken together, recent narrative and empirical work positions the gut microbiome as an actionable interface between ultra-processed dietary exposures and host systems biology in CKM, with microbial signaling, barrier function, and diet–microbe co-metabolism providing convergent pathways that merit targeted measurement and intervention in future CKM-oriented trials, as summarized in Table 2 [9,10,63,64,65,66].

## 7. Practical Design and Methods for Omics-Anchored UPF Interventions

Trials aiming to detect diet-induced multi-omics changes should be powered based on molecular endpoints, not just clinical covariates, as summarized in Table 3. Small, controlled interventions with 20–60 participants can resolve metabolomic and proteomic shifts over 2–8 weeks, with larger cohorts needed for validation [29,30,80]. Longer or heterogeneous interventions require 30–100 participants, with samples taken at baseline and post-intervention [81,82,83,84,85]. These guidelines align with recommendations for well-powered randomized studies, with 20–60 participants for short-term trials and 30–100+ for longer trials [30,80].

Changes in metabolomics and proteomics can be observed quickly, so 2–8 weeks is practical if adherence is high and diets are controlled. Baseline and end sampling usually suffice, with optional early mid-point samples enhancing sensitivity, especially for urine metabolites. Studies show hundreds of metabolites differ with ultra-processed diets, confirming sensitivity to short-term dietary changes [29,30,80]. Microbiome studies often need longer, 6-week to 6-month trials with 30–50 participants, collecting stool at baseline and post-intervention. For durable changes, 3–6 months is recommended [80,81,82]. Controlled trials restricting UPFs report positive taxonomic changes, supporting the proposed timing and sample sizes. Lipidomic and hepatic fat measures change over intermediate periods, requiring 30–70 participants. Changes are more detectable over 8 weeks to 6 months, with measurements at both start and end, especially when aligned with hepatic imaging [83]. Sample collection should match clinical assessments, with rigorous quality control, batch correction (e.g., ComBat, RUV), and randomization to reduce technical noise and conserve study power for a given endpoint [86,87].

## 8. Analytic Rigor: Preventing and Correcting Batch Effects, Controlling Multiplicity, and Assuring Quality

High-throughput diet-omics demands prespecified procedures to minimize variance, correct residual batch effects, control multiplicity, and document quality for reproducibility. It starts with multivariate randomization of samples across plates and days, using tools like Omixer to allocate samples reproducibly and reduce confounding before data collection [88]. This approach best prevents batch artifacts by implementing rigorous pre-analytical controls and documentation, including standardized metadata and biobanking standards to ensure traceability [89].

Residual batch effects, despite prevention, should be detected and corrected using methods such as ComBat, EigenMS, RUV, SERRF, CordBat, RRmix, and tools such as malbacR and dbnorm [86,87,90,91,92,93]. Corrections must be validated with visual and quantitative checks to avoid erasing biological signals. Given the high dimensionality, multiple-testing control using FDR (Benjamini–Hochberg) is essential, with Bonferroni for confirmatory analyses. Predefined hypotheses and power considerations reduce the likelihood of non-replicable findings [46,87,90,92,93,94].

Quality-control pipelines, including pooled materials, standards, and metrics, monitor data reliability and identify low-quality features. Software like OmicsEV aids assessment, while documentation, versioning, and validated software ensure auditability and reproducibility [95,96,97].

## 9. Translational Readiness of Targeted Lipidomic and Proteomic Panels for CKM Risk Stratification and Monitoring

Targeted lipidomic and proteomic assays have advanced, making them suitable as adjunct tools for CKM risk assessment, with biological specificity and technical maturity. Lipid panels that measure ceramides, sphingomyelins, triglycerides, and phospholipids are more available in clinical labs and offer better outcome prediction beyond traditional lipids, supporting links to insulin resistance, atherosclerosis, and kidney disease [98,99,100]. Guidelines recognize targeted omics’ potential to detect early, modifiable risk factors, especially inflammation, mitochondrial dysfunction, and metabolic flexibility [2,75]. These advancements make lipidomics a practical link between mechanistic understanding and risk stratification, though standardization and validation are needed for routine use.

Circulating protein panels offer a complementary way to CKM risk stratification, indexing inflammatory, endocrine, and endothelial pathways not captured by standard markers. Small, assay-ready sets, including proteins such as β-glucuronidase, leptin, aldosterone, soluble neprilysin, and endocan, are measurable with high-throughput assays or targeted mass spectrometry and are prioritized based on reproducible links to CKM traits and events [101,102]. Large cohorts show that proteomic modules reflecting immune activation and vascular dysfunction independently predict coronary heart disease, chronic kidney disease, and mortality, supporting their use as risk markers in research and clinical settings [51]. Table 4 shows the links between higher UPF intake and circulating inflammatory proteins, lipoprotein profiles, adiponectin, complement/coagulation factors, and fibroblast growth factors.

Implementation at scale remains premature. Expert evaluations highlight modest discrimination based on clinical covariates in general populations, with most gains in high-risk groups or near decision thresholds [53,54]. Barriers include platform heterogeneity, incomplete standardization, uncertainty about the actionability of absolute values, and limited validation across ancestries and CKM stages [1,98,115]. Currently, targeted lipidomic and proteomic panels should be used as adjuncts in prospective studies and selected scenarios, while larger validation and cost-effectiveness analyses continue. Routine use in risk calculators and care pathways depends on consistent improvements in prediction, calibration, outcomes, and formal guideline endorsement.

These panels track pathway responses aligned with the AHA’s CKM framework. Lipidomic signatures measure sphingolipid and triglyceride remodeling during interventions, while proteomic modules reflect inflammation and endothelial injury linked to adiposity, glycemia, and kidney function [51,75]. They support secondary endpoints in trials for mechanistic insights alongside clinical risk factors. However, routine monitoring needs standardized procedures, reference materials, and consensus on clinically significant changes.

## 10. Equity and Vulnerability Across Sex, Socioeconomic Status, and Baseline Disease

An equity lens is essential for interpreting heterogeneity in UPF-CKM associations because effect modification aligns with differences in baseline exposure, background risk, and structural constraints. Across studies summarized in this review, both men and women exhibit higher CKM risk with greater UPF intake, although some cohorts report larger associations for obesity and metabolic syndrome among women; these sex differences are not uniformly consistent across populations, and the overall signal remains present in both sexes [14]. Consequently, while biological sex may influence relative risks, the absolute benefit from UPF reduction will depend more on the baseline CKM risk and the degree of UPF exposure within each sex [37,49,116].

Socioeconomic status materially structures exposure and vulnerability. Lower socioeconomic status is consistently linked to higher UPF intake and greater CKM risk independent of other demographic and lifestyle factors, reflecting affordability, accessibility, and marketing environments that concentrate UPFs in disadvantaged communities [14]. These dietary patterns co-occur with higher internal doses of packaging-linked contaminants, such as phthalates and bisphenols, which are elevated among individuals who consume more UPFs and in lower socioeconomic groups, thereby layering additional inflammatory and metabolic stressors on CKM pathways [11,117]. Together, this pattern implies that reducing UPFs in lower socioeconomic strata can yield disproportionately large absolute risk reductions by simultaneously lowering nutrient- and contaminant-related drivers of CKM risk [5,14,49].

Baseline disease status further modifies both susceptibility and potential benefit. Individuals with pre-existing obesity, diabetes, or CKD experience amplified adverse effects of UPFs, including faster CKD progression and higher mortality among those with kidney disease [37,49,116]. Because absolute event rates are highest in these groups, even modest proportional risk reductions from lowering UPFs translate into larger absolute benefits within CKM care pathways that prioritize weight, glycemia, blood pressure, and kidney function. These observations support targeting UPF reduction as part of comprehensive management in high-risk patients while maintaining population-wide guidance.

## 11. Global Context and Population Impact

The adverse association between UPF intake and CKM outcomes is observable across regions with very different food systems. Large multinational cohorts and umbrella reviews report higher mortality and major CKM endpoints with higher ultra-processed food intake in North America, South America, Europe, and Asia, and across urban and rural settings. In the PURE cohort spanning five continents, higher intake of UPFs was associated with greater total and non-cardiovascular mortality, demonstrating that the core signal persists beyond Western dietary contexts, even as its magnitude varies by region [118]. Umbrella reviews that synthesize cohorts from multiple continents have reached similar conclusions, reinforcing the consistency of the association across populations [31,32,119,120,121]. Regional differences in prevalence and trajectory of UPF intake modulate population impact. Intake levels are generally higher in high-income countries; however, consumption is rising rapidly in low- and middle-income countries, where affordability and accessibility make these products common in vulnerable communities [5,49,118]. Furthermore, limited access to healthcare can amplify downstream risks [49]. These contextual factors help explain variation in effect sizes while maintaining the directionality of the association.

The product mix also shapes risk pathways in various food systems. Ultra-processed categories include sugar-sweetened beverages, packaged snacks, processed meats, and ready-to-eat meals, each with distinct nutrient profiles and additives that align with inflammatory and metabolic mechanisms relevant to CKM [122]. Where diets rely more heavily on packaged, ultra-processed items, higher internal doses of packaging-linked contaminants, such as bisphenols and phthalates, have been documented, adding exposure channels that converge on immunometabolic stress [26,27,49,122]. These layers likely contribute to between-region heterogeneity without negating the overall association.

## 12. Conclusions and Research Agenda

UPFs represent a pervasive environmental hazard that accelerates disease progression across the entire cardiovascular-kidney-metabolic (CKM) continuum. While the epidemiological hazard is now robustly established, the field must transition from observational associations to molecularly precise clinical interventions. Multi-omics integration spanning metabolomics, lipidomics, proteomics, and the gut microbiome provides the necessary systems-biology framework to elucidate the specific causal mechanisms (such as meta-inflammation and gut barrier dysfunction) that link UPFs to CKM syndrome.

To advance the scientific understanding and clinical application of UPF exposure within the CKM framework, future research should focus on the following areas: harmonizing exposure metrics by standardizing the use of “percentage of total energy from UPFs” as the main metric in models. This will help minimize systemic under-reporting biases and facilitate accurate comparisons between studies. Additionally, conducting stage-specific, isocaloric randomized controlled trials is essential. These trials, with adequate power, should encompass different AHA CKM stages to clearly differentiate the physiological impacts of food processing from mere macronutrient variations. Standardizing multi-omics workflows is also crucial, involving strict, GLP-aligned bioinformatics pipelines that incorporate proactive multivariate randomization, standardized batch effect correction methods (e.g., ComBat, SERRF), and false discovery rate control to ensure reproducibility in diet-omics data. Furthermore, validating translational risk panels is important; prospective validation of targeted lipidomic and proteomic panels as prognostic tools for CKM risk stratification should be pursued, with the goal of establishing clinically actionable thresholds prior to bedside application.

## Figures and Tables

**Table 1:** Diseases/Conditions Associated with UPF Intake.

Outcome	Study Design	Sample Size	Effect Estimate (95% CI)	Study/Lead Author
Cardiovascular Disease	Prospective Cohort	105,159 participants (NutriNet-Sante cohort)	HR: 1.12 (1.05–1.20)	Srour et al. (2019) [35]
Cardiovascular Events	Meta-analysis	43 studies	RR: 1.04 (1.02–1.06)	Pagliai et al. (2021) [33]
Chronic Kidney Disease (CKD)	Prospective Cohort	78,346 participants (Lifelines cohort)	OR: 1.27 (1.09–1.47)	Cai et al. (2022) [36]
CKD Progression	Prospective Cohort	2616 participants (CRIC study)	HR: 1.22 (1.04–1.42)	Sullivan et al. (2023) [37]
Type 2 Diabetes Mellitus	Prospective Cohort	311,892 participants (EPIC study)	HR: 1.17 (1.14–1.19)	Dicken et al. (2024) [38]
Metabolic Syndrome	Prospective Cohort	8065 participants (ELSA-Brasil cohort)	RR: 1.19 (1.07–1.32)	Canhada et al. (2023) [39]
Obesity and Cardiometabolic	Systematic Review	17 studies	Positive associations reported	Mambrini et al. (2023) [40]
All-cause Mortality	Umbrella Review	9,888,373	RR: 1.21 (1.15–1.27)	Lane et al. (2024) [31]

RR: relative risk, HR: hazard ratio, OR: odds ratio, CI: Confidence Interval.

**Table 2:** Gut microbiome as an actionable interface between UPF exposures and host systems biology in CKM syndrome.

Pathway/Mechanism	Key Microbial Processes and Host Effects	Evidence from Recent Research	Actionable Measurement Targets	Intervention Strategies
Microbial Signaling	Microbial metabolites (e.g., SCFAs, TMAO, LPS) modulate host immune, metabolic, and inflammatory pathways; dysbiosis increases pro-inflammatory signaling and metabolic endotoxemia	UPF-driven dysbiosis elevates TMAO, LPS, and uremic toxins, promoting CKM risk [64,69,70,71]	Circulating TMAO, LPS, SCFA levels; microbial gene/metabolite profiling	Diet modification, pre-/probiotics, FMT, targeted microbial/metabolite therapies
Gut Barrier Function	Dysbiosis and UPFs impair mucosal integrity, increase gut permeability (“leaky gut”), and facilitate translocation of microbial products	Leaky gut and reduced mucus production linked to systemic inflammation and CKM progression [69,71,72,73]	Gut permeability assays, mucosal integrity markers, and inflammatory cytokines	Fiber-rich diets, synbiotics, barrier-protective agents
Diet-Microbe Co-metabolism	Microbes metabolize dietary components (fiber, polyphenols, proteins) into bioactive compounds affecting host metabolism; UPFs reduce beneficial co-metabolism	High-fiber/Mediterranean diets enhance SCFA production, improve metabolic resilience; UPFs reduce microbial diversity and beneficial metabolites [74,75,76,77]	Microbial diversity, SCFA profiles, metagenomics/metabolomics	Precision nutrition, personalized dietary interventions
Systems Biology Integration	Gut microbiome acts as a hub for the gut-heart-kidney axis, mediating inter-organ signaling and homeostasis	Systems biology frameworks highlight remote sensing/signaling and organ cross-talk via microbial metabolites [64,71,75]	Multi-omics (metagenomics, metabolomics, transcriptomics), network analysis	Integrated multi-target interventions, biomarker-guided trials
Future Clinical Trial Targets	Convergent pathways: microbial signaling, barrier integrity, co-metabolism; need for targeted measurement and intervention	Narrative and empirical research advocate for measuring microbial metabolites, barrier function, and diet-microbe interactions in CKM trials [71,75,78,79]	Composite endpoints: metabolite panels, barrier markers, clinical CKM outcomes	Multi-modal interventions (diet, microbiome modulation, systems biology-guided therapies)

SCFA: short-chain fatty acid, TMAO: trimethylamine N-oxide, LPS: lipopolysaccharide, FMT: fecal microbiota transplantation.

**Table 3:** Sample sizes and timepoints required to detect meaningful changes in multi-omics endpoints in UPF-reduction RCTs.

Omics Layer	Typical Sample Size	Intervention Duration	Key Timepoints	References
Metabolomics	20–60 (short-term); 100+ (validation)	2–8 weeks (feeding); 6–12 months (cohort)	Baseline, end-of-phase; optional midpoint	[29,30,80]
Proteomics	20–60	2–8 weeks	Baseline, end-of-phase	[30,80]
Microbiome	30–50 (RCT); 70+ (long-term)	6 weeks–6 months	Baseline, post-intervention	[81,82,83,84]
Lipidomics	30–70	8 weeks–6 months	Baseline, end-of-phase	[80,85]
Hepatic Fat (MRI)	30–70	6 months	Baseline, post-intervention	[85]

MRI: magnetic resonance imaging, RCT: randomized controlled trial.

**Table 4:** Associations between higher UPF intake and circulating protein concentrations.

Protein/Marker	General Change with Higher UPF Intake	Population Consistency	References
CRP/hs-CRP	↑ Robustly higher	Adults, adolescents	[103,104,105,106]
IL-6	↑ Higher	Adults	[103,107,108,109]
TNF-α	↑ Higher	Adults	[103,107,108,109]
Leptin	↑ Higher	Adults, adolescents	[106,108]
IL-8, IL-15	↑ Higher	Adolescents, older adults	[106,108]
ApoB	↑ Higher	Adults	[110,111]
HDL, LDL subclasses	↓ HDL, smaller LDL/HDL size	Adults	[111]
Adiponectin	↓ Lower (less consistent)	Adults with obesity	[112]
Resistin	↑ Higher (adiposity-related)	Adults with obesity	[109,112]
PAI-1, complement	↑ Limited/inconsistent	Adults	[51,103,109]
FGF-19	↓ Lower	Adults	[113]
FGF-21	↑ Acute (↑ with sugar, ↓ with protein)	Healthy adults	[47,114]

CRP: C-reactive protein, hs-CRP: high-sensitivity CRP, IL: interleukin, TNF: tumor necrosis factor, Apo: apolipoprotein, HDL: high-density lipoprotein, LDL: low-density lipoprotein, PAI: plasminogen activator inhibitor, FGF: fibroblast growth factor, ↑: increase, ↓: decrease.

## Data Availability

This study is a narrative review based on previously published literature. No new data were created or analyzed in this work. Therefore, data availability is not applicable.

## Abbreviations

AHA	American Heart Association
ApoB	Apolipoprotein B
BMI	Body mass index
ELSA-Brasil	Brazilian Longitudinal Study of Adult Health
CVD	Cardiovascular disease
CKD	Chronic kidney disease
ComBat	Combining batches
CordBat	Concordance-based batch effect correction
dbnorm	Dataset batch normalization
EigenMS	Eigen-analysis of Mass Spectrometry
EPIC	European Prospective Investigation into Cancer and Nutrition
FDR	False discovery rate
FGF-19, FGF-21	Fibroblast growth factor-19, -21
GLP	Good laboratory practice
HDL	High-density lipoprotein
Hs-CRP	High-sensitivity C-reactive protein
IL-6, IL-8, IL-15	Interleukin-6, -8, -15
KDIGO	Kidney Disease: Improving Global Outcomes
LPS	Lipopolysaccharides
LDL	Low-density lipoprotein
MRI	Magnetic resonance imaging
PAI-1	Plasminogen activator inhibitor-1
RRmix	Random main effect and random compound-specific error variance with a mixture structure
RUV	Remove unwanted variation
SCFAs	Short-chain fatty acids
SERRF	Systematic error removal using random forest
TMAO	Trimethylamine N-oxide
TNF-α	Tumor necrosis factor alpha
UPF	Ultra-processed food
